# Psychedelic minimalism: the case against music in psychedelic therapy settings

**DOI:** 10.3389/fpsyt.2025.1652568

**Published:** 2025-08-26

**Authors:** Samir A Nader

**Affiliations:** Macon & Joan Brock Virginia Health Sciences at Old Dominion University Eastern Virginia Medical School, Norfolk, VA, United States

**Keywords:** psychedelic therapy, set and setting, music intervention, emotional modulation, non-ordinary states of consciousness, psychedelic minimalism, introspection, therapeutic environment

*In response to:* Kaelen et al. (1)*, “The Hidden Therapist: Evidence for a Central Role of Music in Psychedelic Therapy.”*

## Introduction: reframing the role of setting

In recent years, renewed attention has been given to the centrality of *set and setting* in psychedelic-assisted therapy. The Reporting of Setting in Psychedelic Clinical Trials (ReSPCT) guidelines reflect an evolving consensus: therapeutic outcomes are not solely pharmacologically determined but are heavily shaped by both the environment and the psychological state of the participant (2). Within this evolving paradigm, music has assumed a privileged role, framed as a therapeutic constant, typically curated to be emotionally supportive or evocative, and especially emphasized during peak psychedelic states (3). The prevailing assumption is that music facilitates safety, emotional access, and therapeutic alignment (1). However, this assumption has not been subjected to the same degree of empirical or philosophical scrutiny that other elements of the setting have received.

In this opinion, I offer a contrarian view: that the inclusion of music in psychedelic therapy may not be benign or beneficial but could instead function as a confounding variable, altering, distorting, or displacing the very psychological material that therapy seeks to access. I argue that music introduces external emotional content that may obscure the patient’s natural psychological flow, their *set*, thereby undermining the core objective of psychedelic therapy: unmediated engagement with the self. This perspective, which I call *psychedelic minimalism*, challenges the default assumption that additive features of the setting, particularly music, are inherently therapeutic. Rather than continuing to ask what kind of music, or how it’s played, best supports the psychedelic experience, I suggest we ask a more foundational question: why music at all? If the therapeutic goal is to access internal, unfiltered psychological material, then any emotionally potent stimulus, no matter how well-curated, may interfere with that process. I also call for further research to fill this gap, exploring whether a minimalist setting, free of emotional modulation, offers deeper, more authentic access to the self.

## Setting, set, and the risk of emotional interference

In psychedelic therapy, *set* refers to the totality of a patient’s internal state, their beliefs, thoughts, personality, and emotional tendencies, while *setting* denotes the external environment with its sensory, and interpersonal dimensions (4). Together, set and setting shape the content, tone, and trajectory of the psychedelic experience (5).

Psychedelics generate non-ordinary states of consciousness (NOSCs) not inherently therapeutic in themselves but filled with potential emerging from lowered psychological defenses, allowing dissociated material to surface (6). When this material is engaged with, the therapeutic action of psychedelics obtains; the role of the setting should not be to guide the patient away from this material, but to allow its unfolding with as little interference as possible allowing for direct engagement.

To that end, the ideal therapeutic setting is emotionally neutral, a vessel that supports the emergence of unfiltered psychological content without adding its own emotional coloration. I argue that psychedelic therapeutic efficacy hinges most critically on preserving the primacy of the set, enabling the individual to encounter their psyche without distortion or displacement from external stimuli; namely, that the goal of what I refer to as the *pure set* encounter is a setting designed to unconfound the set, where every additive feature of the setting is evaluated for how it alters the natural flow of cognition and introspection.

## Music as a modulator of emotion and meaning

Among all components of the therapeutic setting, music stands out as a uniquely potent psychological force. It is not ambient noise or passive background, rather, it is emotionally loaded, temporally structured, and rich with meaning (7, 8). Music in psychedelic therapy is often justified for its ability to guide, ground, or emotionally stabilize the patient during challenging experiences (1). Yet it is this very power that warrants caution.

I propose that music operates as a *phenomenological modulating influence*, shaping the patient’s emotional and cognitive flow during psychedelic sessions. Clinical data and emerging theories in affective neuroscience and music therapy suggest two primary axes by which music may exert its effects: emotional provocativeness, or the ability to evoke strong affective states like awe, nostalgia, grief, or anxiety, and personal meaning, referring to the autobiographical associations embedded within particular songs (3, 9, 10). Music scoring high on either dimension, let alone both, I argue, risks overpowering the patient’s natural stream of thought and redirecting attention to emotionally charged content that may not have otherwise emerged.

The influence of provocative music has been observed clinically and acknowledged even in the ReSPCT Guidelines (Item 11), which call for careful reporting of the music used, given its evident impact on the therapeutic setting (11). One case study participant described her psilocybin session as uncomfortable when accompanied by music, but calming and insightful during silent periods that involved therapist-guided mindfulness and interpersonal discussion. She ultimately chose to move to a quiet room, preferring a music-free setting (12). Another participant stated that “under psilocybin I felt almost that I had no choice but to go with the music” (1), implying a surrender of agency to the external structure of sound. These accounts suggest that music, far from universally supportive, can inhibit introspection and redirect the therapeutic process away from self-generated insight.

Proponents of music often argue that it enhances the psychedelic experience and supports patients through difficult terrain (13). But this assumption is worth challenging. In many cases, meaningful breakthroughs occur not through comfort but through productive discomfort: the slow processing of unstructured inner chaos, the confrontation with long-avoided emotions, or the resurfacing of deeply repressed memory. One psilocybin trial participant described her musical experience as overwhelming, reporting that she felt she was “suffocating,” and that the music was “too much” (12). Only during a music-free period did she experience release from resistance and begin productive introspection.

Even music that holds personal significance may be counterproductive. A song tied to the memory of a lost loved one may elicit genuine sadness, but that sadness is structured by the music’s associative framework, not by the patient’s internal emotional terrain. While some may argue that such triggers can facilitate emotional processing, especially when linked to trauma, the ability to identify and select such music in advance is clinically uncertain and not scalable. Many patients are unaware of how particular songs will affect them until they are in the altered state. Furthermore, music that appears helpful in one moment may overwhelm or displace more relevant material that would otherwise emerge in silence. The risk here is not just distraction, but *displacement*: the substitution of an externally prompted emotional script for an internally driven exploration.

## Gaps in research, future directions, and analysis of conflicts of interest

Despite the central role music plays in most psychedelic-assisted therapy protocols, there is a striking lack of controlled research examining its specific effects. Music is often treated as a default or essential component, not as a testable variable. This has left a significant gap in our understanding of how therapeutic outcomes differ between music-enriched and minimalist (silent) settings. The assumption of music’s benefit remains largely unchallenged, limiting insight into which aspects of the psychedelic experience are truly intrinsic and which are shaped by external influences.

This oversight presents a valuable opportunity for future research. Clinically testable questions, such as whether music fosters emotional insight or deflects it, and whether insights gained in silence are more enduring, could inform more effective, scalable treatment models. Randomized trials, qualitative and neurophenomenological studies, and long-term follow-ups comparing different settings could clarify whether music enhances or interferes with therapeutic goals. Rather than accepting tradition, researchers must begin to treat music as an active variable whose inclusion requires empirical justification.

It is also not without mention, notable to consider the potential conflict between author Mendel Kaelen, PhD, the first author of the dissented paper, and his role as CEO of Wavepaths, a “startup providing music both for and as psychedelic therapy” (14, 15). If Dr. Kaelen has a financial stake in the use of music in the setting of psychedelic assisted therapy, this invites scrutiny of potential influence over confirmation bias in his research, especially given the nascent, subjective nature of current psychedelic medicine research.

## Discussion

Psychedelic therapy is most powerful when it enables unmediated access to the self. Yet music, widely assumed to be therapeutic, may disrupt this process by directing attention and shaping emotional response. As a modulating force, it risks overshadowing the very psychological material therapy aims to access. Emotional experiences prompted by music may feel meaningful but are often structured by the music itself, not by the patient’s own psyche. A minimalist setting that is quiet, neutral, and non-directive, offers an alternative. Rather than guiding or soothing, it allows internal content to emerge undisturbed. If the goal is authentic introspection and lasting transformation, then silence may be more supportive than sound. Future research should directly test this, shifting the burden of proof onto any setting feature, especially music, that claims therapeutic value.
