# Supplementary material for: Psychedelic minimalism: the case against music in psychedelic therapy settings
Source: Front Psychiatry. 2025 Aug 26;16:1652568. doi: 10.3389/fpsyt.2025.1652568 (PMC12417490; doi:10.3389/fpsyt.2025.1652568)
Supplement: Supplementary file 1 [file Table1.docx]

Supplementary file – Generative AI Initial and final prompts

**Initial Prompt**

*“I am working on an opinion article about the role of music in psychedelic therapy settings. Please provide information on how music might act as a confounder, the role of set and setting in psychedelic therapy, and any relevant research or citations that could support a minimalist approach to the therapeutic environment.”*

**Final Prompt**

*“Please do a final check of my manuscript, ensure grammar and clarity, confirm that all claims are supported by valid citations, and generate appropriate keywords for submission.”*
